# Maintaining human fetal pancreatic stellate cell function and proliferation require β1 integrin and collagen I matrix interactions

**DOI:** 10.18632/oncotarget.4338

**Published:** 2015-06-02

**Authors:** Bijun Chen, Jinming Li, George F. Fellows, Zilin Sun, Rennian Wang

**Affiliations:** ^1^ Department of Endocrinology, Zhongda Hospital, Institute of Diabetes, Medical School, Southeast University, Nanjing, Jiangsu, China; ^2^ Children's Health Research Institute, University of Western Ontario, London, ON, Canada; ^3^ Department of Physiology and Pharmacology, University of Western Ontario, London, ON, Canada; ^4^ Department of Obstetrics and Gynecology, University of Western Ontario, London, ON, Canada

**Keywords:** human fetal pancreatic stellate cells, integrins, extracellular matrix, signaling pathway

## Abstract

Pancreatic stellate cells (PaSCs) are cells that are located around the acinar, ductal, and vasculature tissue of the rodent and human pancreas, and are responsible for regulating extracellular matrix (ECM) turnover and maintaining the architecture of pancreatic tissue. This study examines the contributions of integrin receptor signaling in human PaSC function and survival. Human PaSCs were isolated from pancreata collected during the 2^nd^ trimester of pregnancy and identified by expression of stellate cell markers, ECM proteins and associated growth factors. Multiple integrins are found in isolated human PaSCs, with high levels of β1, α3 and α5. Cell adhesion and migration assays demonstrated that human PaSCs favour collagen I matrix, which enhanced PaSC proliferation and increased TGFβ1, CTGF and α3β1 integrin. Significant activation of FAK/ERK and AKT signaling pathways, and up-regulation of cyclin D1 protein levels, were observed within PaSCs cultured on collagen I matrix. Blocking β1 integrin significantly decreased PaSC adhesion, migration and proliferation, further complementing the aforementioned findings. This study demonstrates that interaction of β1 integrin with collagen I is required for the proliferation and function of human fetal PaSCs, which may contribute to the biomedical engineering of the ECM microenvironment needed for the efficient regulation of pancreatic development.

## INTRODUCTION

Pancreatic stellate cells (PaSCs) are non-endocrine, mesenchymal-like cells [1-3] that reside in the peri-acinar, peri-ductal and peri-vascular area and play a role in regulating extracellular matrix (ECM) turnover, which is important for maintaining the integrity of pancreatic tissues architecture [4]. In the adult human pancreas, there is emerging evidence that interplay between PaSCs and the surrounding ECM is required for pancreatic tissue repair and involved in pathophysiological processes including inflammation and pancreatic fibrosis [5]. However, little is known about the interaction between PaSCs integrin receptors and various ECM components in the developing human pancreas.

Integrins are cell adhesion receptors that play an integral role in cell-cell and cell-ECM communication in many cell types, including rodent PaSCs. Among integrin receptors, β1 integrin associates with 12 α subunits [6] and mediates cellular binding to multiple extracellular matrix proteins (i.e., collagens, laminin, fibronectin) [7]. Stimulation of β1 integrin down-stream signaling pathways are important for cell migration, differentiation, proliferation, and survival [7-9]. Our recent study elucidated the functional role of β1 integrin in PaSCs using an inducible β1 integrin knockout mouse model [10]. We found that β1 integrin deficiency in collagen I-producing PaSCs impaired basement membrane integrity and function with loss of ECM expression in the pancreas, which in turn affected acinar cell proliferation and function [10]. The connective tissue growth factor (CTGF), a known activator of PaSCs, requires α5β1 integrin to promote rat PaSCs adhesion, migration, proliferation, and cytokine secretion [11]. In the developing human pancreas, the α3β1 integrin is highly expressed in fetal islet cells and co-localizes with collagen IV [12]. Interactions between α3β1 and collagen I or IV activate both the MAPK/ERK and the PI3K/AKT signaling pathways, suggesting that they are critical for modulating islet cell differentiation, proliferation, and survival in the developing human pancreas [13]. Despite these previous studies, the functional role of β1 integrin directly affecting PaSCs in the developing human pancreas has yet to be examined.

In this study, PaSCs were isolated from human fetal pancreata collected during the 2^nd^ trimester of pregnancy and characterized as follows: the expression pattern of β1 integrin associated with α subunits in human PaSCs were examined, and cell behaviors such as adhesion, migration, proliferation, and function were studied following exposure to various matrix proteins. Our results revealed that the collagen I matrix increased α3β1 integrin-dependent PaSC adhesion and migration, enhanced PaSC proliferation, and increased TGFβ1 and CTGF production via activation of the FAK/ERK and AKT signaling pathways. Similar results were obtained when using a β1 integrin blockade study. This study demonstrated that β1 integrin association with α3 integrin is required for the proliferation and proper function of human fetal PaSCs on a collagen I matrix, and provide a better understanding of PaSC-ECM interactions in the developing human pancreas.

## RESULTS

### Characterization of purified human fetal PaSCs

**Figure 1:**
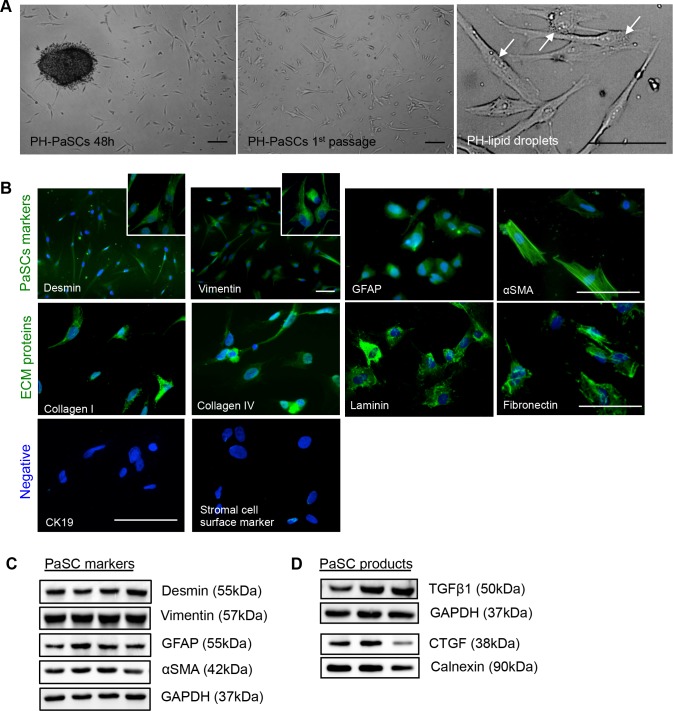
Characterization of isolated human fetal PaSCs **A.** Phase-contrast micrographs of quiescent human fetal PaSCs. Scale bar: 50μm. Arrows indicate lipid droplets in PaSCs. **B.** Representative images of immunofluorescence staining for PaSC markers: desmin, vimentin, GFAP, and αSMA, along with ECM proteins: collagen I and IV, laminin, and fibronectin (green). Both CK19 (ductal cell marker) and stromal cell surface marker were not expressed in the human fetal PaSCs. Nuclei were stained with DAPI (blue). Scale bar: 50μm. Magnified images for desmin and vimentin are shown in the insets. Western blot analyses of PaSC markers **C.** and growth factors (TGFβ1 and CTGF) **D.** from cultured human fetal PaSCs, between passages 2 to 5 (*n* = 5-6 experiments/group). Representative blots are shown.

Using a modified outgrowth method [14], human fetal PaSCs were outgrown to form monolayers within 3 weeks of the culture, where the phase-contrast (PH) micrograph of quiescent PaSCs for lipid droplet content was used to confirm PaSC phenotype (Figure 1A). The purity of PaSCs was assessed by immunofluorescence staining and western blot analysis, and showed positive labeling for stellate cell selective markers (desmin, vimentin, GFAP, and αSMA), but not for cytokeratin 19 (a ductal cell marker) and stromal cell surface marker (Figure 1B). The matrix proteins, collagen I and IV, laminin, and fibronectin, were also expressed in isolated human PaSCs (Figure 1B). Western blot analyses further verified PaSCs markers (Figure 1C), and growth factors (TGFβ1 and CTGF) in cultured human PaSCs from 2 to 5 passages (Figure 1D). This data indicates that purified PaSCs isolated from human fetal pancreata could be used for the following experiments.

### Integrin expression in human fetal PaSCs

**Figure 2:**
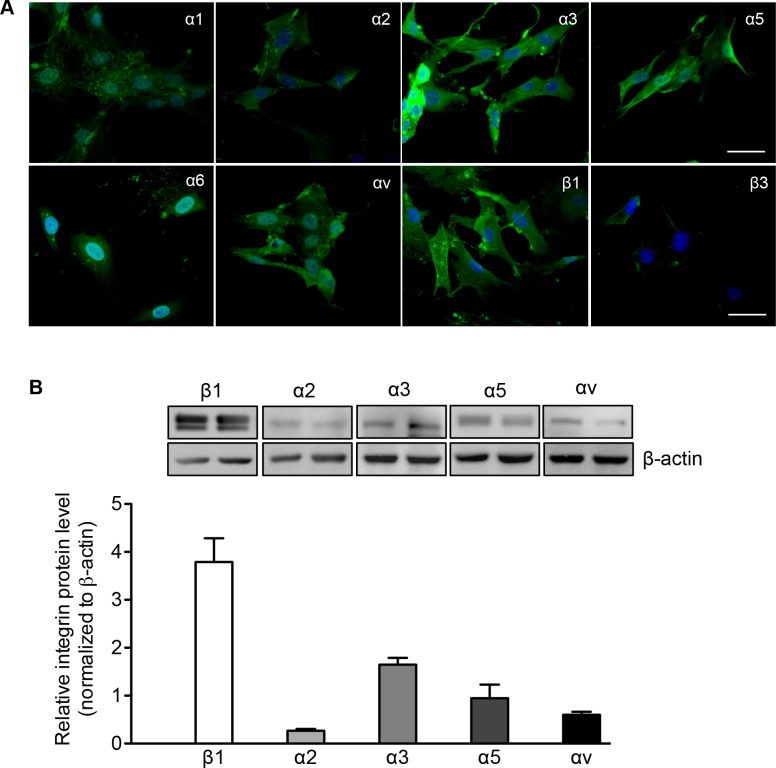
Integrin expression in human fetal PaSCs **A.** Representative images of immunofluorescence staining for α1-3, α5-6, αv, β1 and β3 integrin (green) in cultured human fetal PaSCs. Nuclei were stained with DAPI (blue). Scale bar: 50μm. **B.** Western blot of α and β1 integrin expression in cultured human fetal PaSCs. Data are expressed as means ± SEM (*n* = 5-6 experiments/group). Representative blots are shown.

β1 and β3 integrins and their associated α subunits, including α1-3, α5-6, and αv, were examined in isolated human PaSCs. Immunofluorescence staining of PaSCs showed the expression of α1-3, α5-6 and αv subunits along with β1 integrin, with relatively lower expression of β3 (Figure 2A). The staining data was corroborated with western blot analysis that showed higher levels of β1 integrin with α3 and α5 subunits (Figure 2B). These data suggest that α3β1 and α5β1 may play an integral role in the function and proliferation of human fetal PaSCs.

### Collagen I, IV, and fibronectin matrix proteins enhance PaSC adhesion and migration

**Figure 3:**
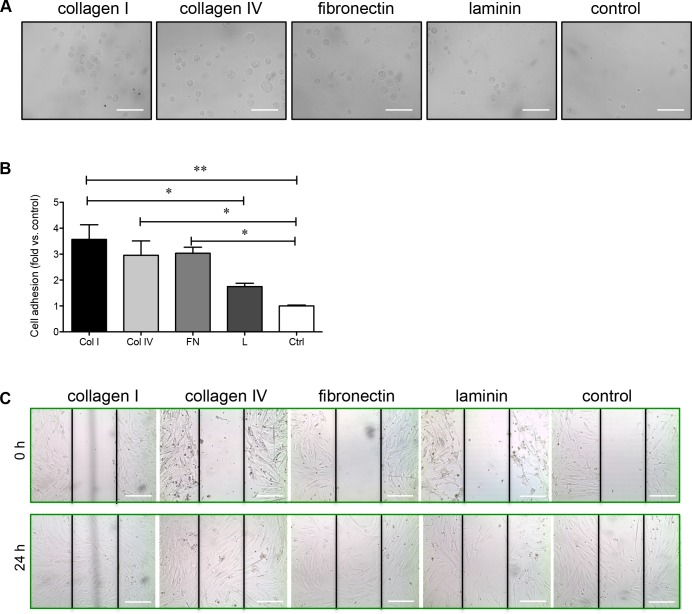
Collagen I, collagen IV, and fibronectin enhance PaSC adhesion and migration **A.** Phase-contrast micrographs of human fetal PaSCs plated on collagen I (Col I), IV (Col IV), fibronectin (FN), laminin (L) or BSA coated control (Ctrl) for 20 mins. **B.** The cell adhesion rate on various ECM matrices after 20 minutes. Data are expressed as means ± SEM (*n* = 4 experiments/group). *p < 0.05, **p < 0.01 vs. control, and analyzed by one-way ANOVA followed by Tukey's post-hoc analyses. **C.** Phase-contrast micrographs of human fetal PaSC migration from wounded gaps at 0 and 24 hours on various ECM matrices. Scale bar: 100μm. Representative images are shown.

The common ligands for α3β1 integrin are fibronectin, laminin, collagen I, and collagen IV [15, 16], while fibronectin is the ligand for α5β1 integrin [17-19]. The adhesion of PaSCs to these common matrix components was examined individually. PaSCs showed strong adhesion to collagen I, collagen IV, and fibronectin within 20 minutes (Figure 3A), and the number of adherent cells increased by at least 3-fold with these matrix components compared to control (*p* < 0.05-0.01, Figure 3B). PaSCs plated on laminin showed relatively lower adhesion when compared to PaSCs on collagen I (*p* < 0.05, Figure 3B), with no significant difference compared to control (Figure 3B). To examine PaSC migration on these matrices, near confluent cell monolayers were wounded to form a gap and migration distance over the gap of PaSCs was measured after 24 hours. It was found that PaSC migration was enhanced on both collagens I and IV, and the gaps were closed completely within 24 hours (Figure 3C). However, gaps remained open on fibronectin, laminin, and control groups (Figure 3C), suggesting that human fetal PaSC migration is favored when cultured on collagen matrices.

### Evaluation of the effect of collagen I on PaSC proliferation

**Figure 4:**
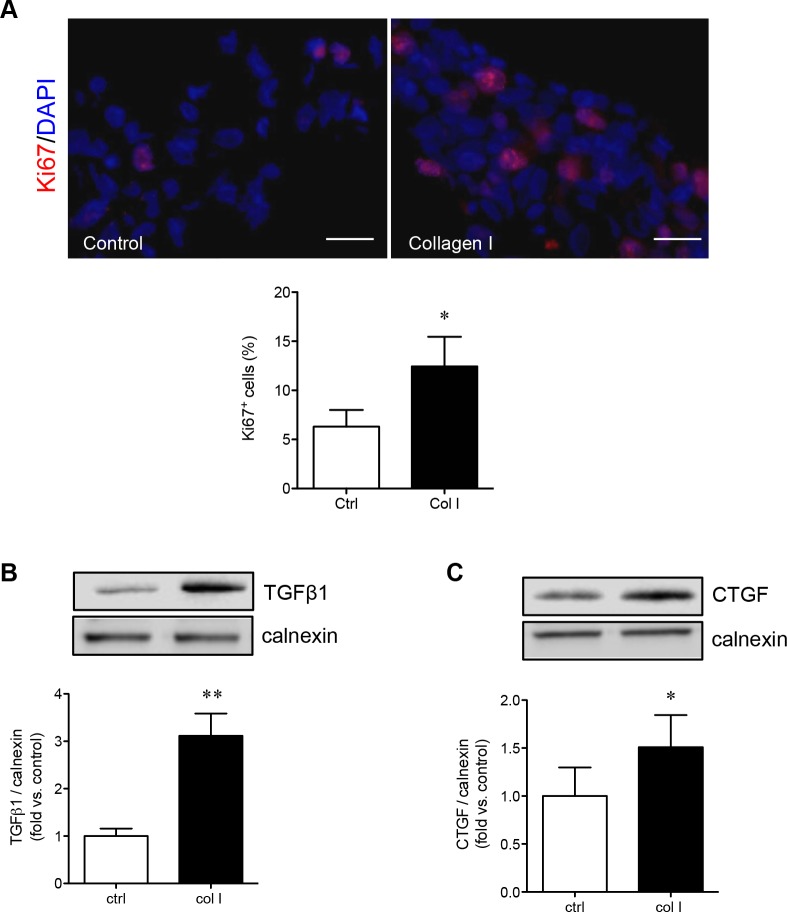
Collagen I increases PaSC proliferation and growth factor production **A.** Representative images of immunofluorescence staining and quantitative analysis of Ki67^+^ (red) for PaSCs cultured on a control plate with BSA or collagen I matrix for 24 hours. Nuclei were stained with DAPI (blue). Scale bar: 25μm. Western blot analyses of TGFβ1 **B.** and CTGF **C.** protein levels in PaSCs cultured on control plate with BSA or collagen I matrix after 24 hours. Representative blots are shown. Data are expressed as means ± SEM (*n* = 4-5 experiments/group). *p < 0.05, **p < 0.01 vs. control, and analyzed by paired Student's *t*-test.

Given that PaSCs cultured on collagen I demonstrated increased PaSC adhesion and migration, the proliferation, growth factors productions (i.e., TGFβl and CTGF) and associated signaling pathways were examined. The proliferative capacity of PaSCs was analyzed by immunofluorescence staining for nuclear Ki67 labeling (Figure 4A), and showed a significantly increased percentage of Ki67^+^ PaSCs when compared to control (*p* < 0.05, Figure 4A). Both TGFβ1 and CTGF are key growth factors produced by PaSCs that stimulate the synthesis and secretion of ECM proteins (collagen, fibronectin, and laminin) [20, 21], and were significantly increased (TGFβ1, *p* < 0.01, Figure 4B; CTGF, *p* < 0.05, Figure 4C) in PaSCs when compared to the control group.

**Figure 5:**
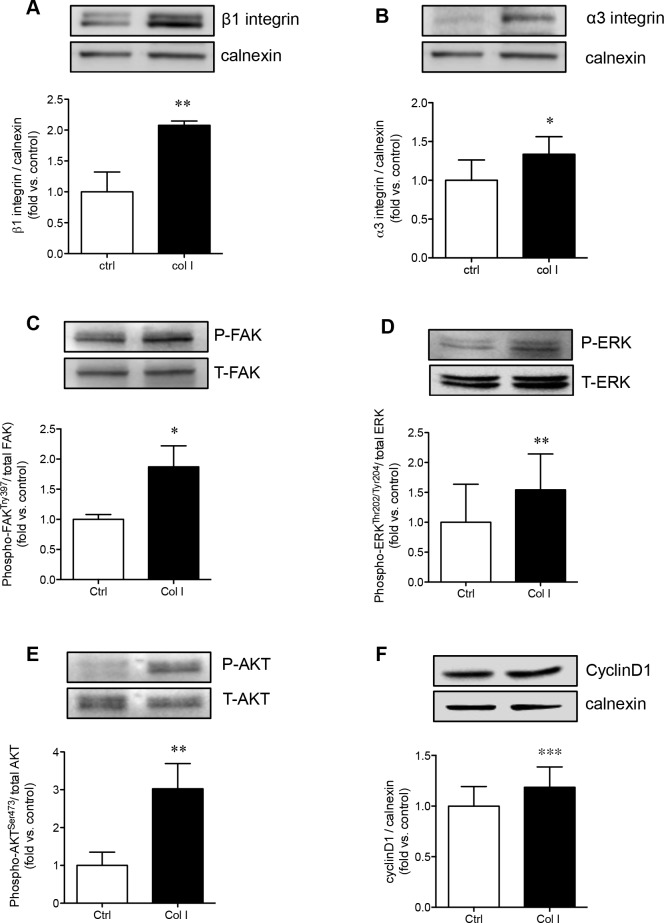
Collagen I enhances PaSC α3β1 integrin expression and downstream FAK/ERK, AKT, and cyclin D1 signaling Western blot analyses of β1 **A.** and α3 **B.** integrins, phosphorylated [P] and total [T] FAK **C.**, ERK **D.**, AKT **E.**, and cyclin D1 **F.** protein levels of PaSCs cultured on a control plate with BSA or collagen I matrix after 24 hours. Data are expressed as means ± SEM (*n* = 4-5 experiments/group). Representative blots are shown. *p < 0.05, **p < 0.01 vs. control, and analyzed by paired Student's *t*-test.

Collagen I is a major activator of α3β1 integrin, and therefore was further examined to determine if PaSCs cultured on collagen I could enhance α3 and β1 integrin expression and stimulate its downstream signaling molecules involved in PaSC proliferation and function. It was noted that both β1 (*p* < 0.01, Figure 5A) and α3 (*p* < 0.05, Figure 5B) protein levels were significantly increased when PaSCs were cultured on collagen I, along with elevated phospho-FAK (*p* < 0.05, Figure 5C) compared to the control group. Increasing activation of α3β1/FAK resulted in a significant increase of phospho-ERK1/2 (*p* < 0.01, Figure 5D) and phospho-AKT (*p* < 0.01, Figure 5E), along with an increase of cyclin D1 (*p* < 0.01, Figure 5F) protein expression, in PaSCs. This data indicates that interaction of α3β1 integrin with collagen I could lead to activation of downstream FAK/ERK and AKT, along with cyclin D1 signaling pathways, resulting in increased PaSC proliferation and function.

### Blocking β1 integrin reduces collagen I stimulated PaSC function and proliferation

**Figure 6:**
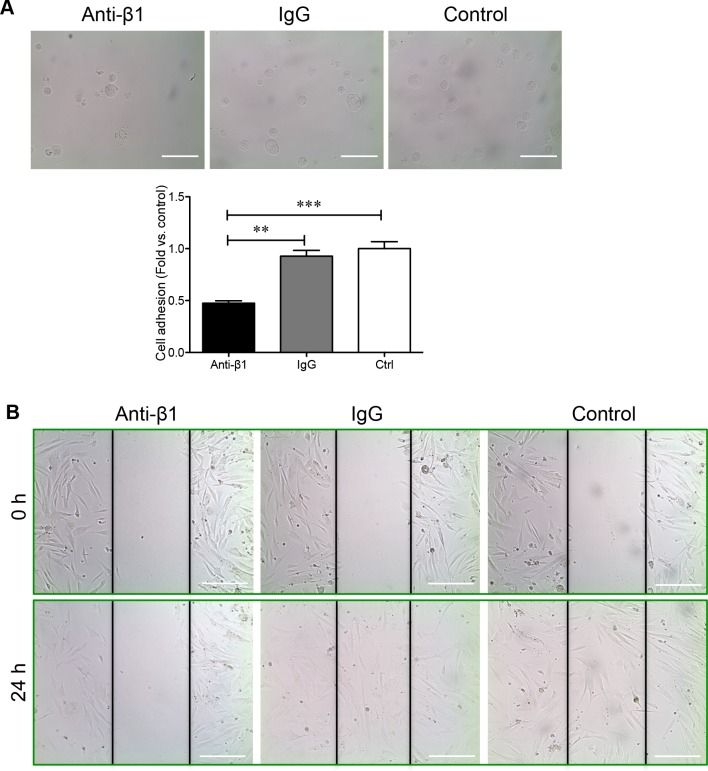
β1 integrin blockade reduces PaSC adhesion and migration on collagen I **A.** Phase-contrast micrograph of human fetal PaSC adhesion and quantitative analyses of PaSC adhesion rate in anti-β1 integrin, IgG, and control experimental groups cultured on a collagen I matrix after 20 minutes. Data are expressed as means ± SEM (*n* = 3 experiments/group). *p < 0.05, **p < 0.01 *vs*. control, and analyzed by one-way ANOVA followed by Tukey's post-hoc analyses. **B.** Phase-contrast micrographs of human fetal PaSC migration from wounded gaps at 0 and 24 hours on collagen I matrix. Scale bar: 100μm. Representative images are shown.

**Figure 7:**
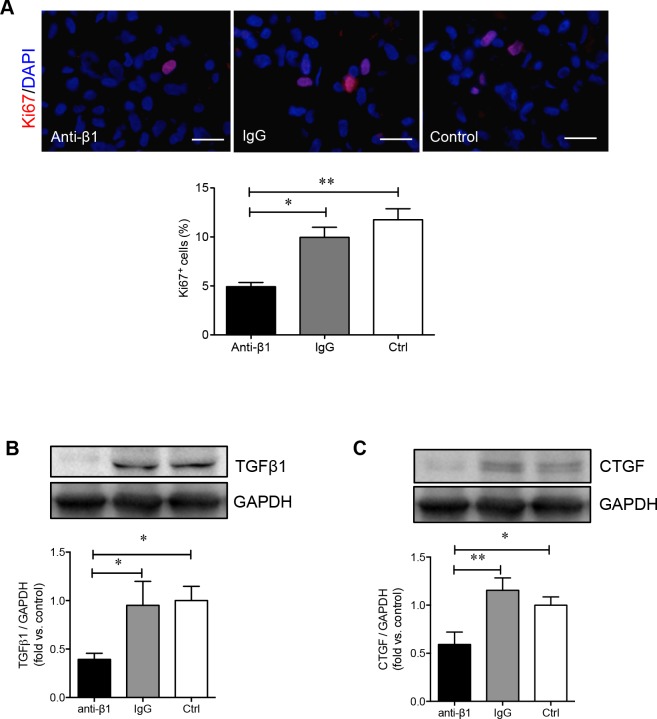
β1 integrin blockade decreases PaSC proliferation and associated growth factor levels **A.** Representative images of immunofluorescence staining and quantitative analyses of Ki67^+^ (red) PaSCs in anti-β1 integrin, IgG and control experimental groups cultured on a collagen I matrix for 24 hours. Nuclei were stained with DAPI (blue). Scale bar: 25μm. Western blot analysis of PaSC TGFβ1 **B.** or CTGF **C.** protein levels in anti-β1 integrin, IgG, and control experimental groups cultured on collagen I for 24 hours. Representative blots are shown. Data are expressed as means ± SEM (*n* = 3-5 experiments/group). *p < 0.05 and **p < 0.01 vs control group, and data were analyzed by one-way ANOVA followed by Tukey's and Fisher's least significant difference post-hoc analyses.

To determine how β1 integrin is involved in regulating the adhesion and migration of PaSCs via the binding of collagen I, human fetal PaSCs were pretreated with human β1 immunoneutralizing antibody, IgG, or left untreated and plated on a collagen I matrix. PaSCs treated with anti-β1 integrin displayed a 50% reduction in cell adhesion to collagen I when compared with IgG and control groups, respectively (*p* < 0.01-0.001, Figure 6A). Functional blockade of β1 integrin on PaSCs severely hampered their ability to migrate and cover the gaps on collagen I matrix (Figure 6B). No apparent difference in PaSC adhesion and migration was observed between IgG and control groups (Figure 6A and 6B). A significant decrease of Ki67^+^ labeling in PaSCs cultured on a collagen I matrix was observed following anti-β1 integrin treatment when compared to controls (*p <* 0.01, Figure 7A). Furthermore, blocking β1 integrin on PaSCs showed relatively reduced TGFβ1 (*p <* 0.05, Figure 7B) and CTGF (*p <* 0.05-0.01, Figure 7C) protein levels when compared to the controls. This β1 integrin blocking study indicates that β1 integrin interactions with the collagen I are essential for maintaining human fetal PaSC adhesion, migration, proliferation, and production of certain growth factors.

### Perturbing β1 integrin function decreases FAK and ERK1/2, but not AKT activation in PaSCs cultured on a collagen I matrix

**Figure 8:**
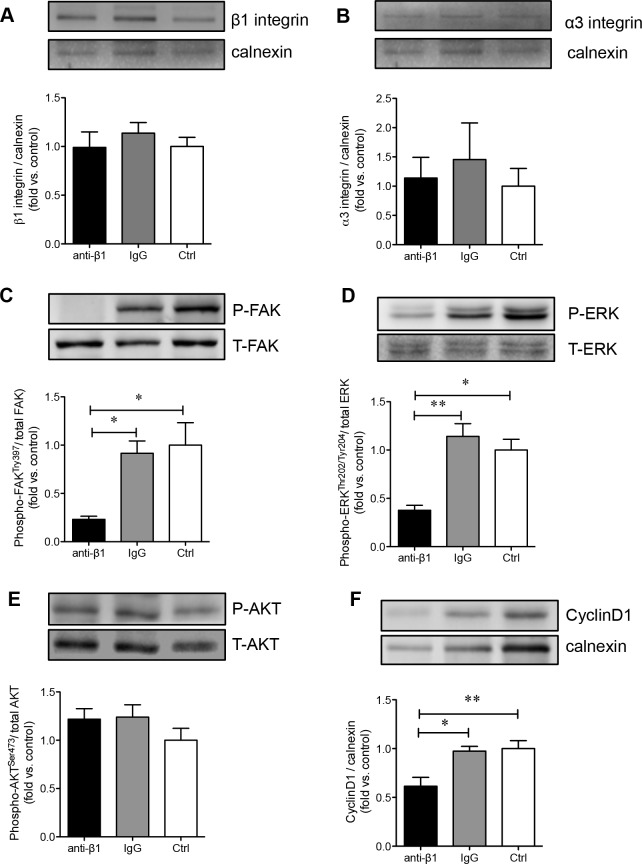
β1 integrin blockade decreases FAK, ERK1/2 and cyclin D1 signaling in PaSCs Western blot analyses of β1 **A.** and α3 **B.** integrins, phosphorylated [P] and total [T] FAK **C.**, ERK **D.**, AKT **E.**, and cyclinD1 **F.** protein levels of PaSCs cultured on collagen I in anti-β1 integrin, IgG, and control experimental groups. Data are expressed as means ± SEM (*n* = 3-5 experiments/groups). Representative blots are shown. *p < 0.05 and **p < 0.01 vs control group, and analyzed by one-way ANOVA followed by Tukey's post-hoc analyses.

Since collagen I promoted PaSC proliferation and production of TGFβ1 and CTGF via the α3 and β1 integrin-stimulated MAPK and PI3K pathways, we examined whether blocking β1 integrin impeded PaSC function and survival via the same pathways. Blocking β1 integrin on PaSCs cultured on a collagen I matrix did not affect the protein levels of β1 (Figure 8A) and α3 (Figure 8B) integrin. However, a significant reduction in the phosphorylation of FAK (*p* < 0.05, Figure 8C) and phospho-ERK (*p* < 0.05-0.01, Figure 8D), but not AKT (Figure 8E), signaling pathways was observed in the anti-β1 PaSC group. This data corroborates with our previous study using human fetal islet-epithelial cells [13]. Additionally, down-regulation of FAK/ERK signaling pathways in PaSCs induced by β1 integrin blockade led to reduced cyclin D1 expression (*p* < 0.05-0.01, Figure 8F). This data further indicates that β1 integrin-collagen I interactions are critical for modulating PaSC proliferation and function through specialized signaling cascades.

## DISCUSSION

In the present study, we have isolated and purified human PaSCs from developing pancreata and have demonstrated that β1 integrin and its associated α3 subunit is highly expressed in human fetal PaSCs. The activation of α3β1 integrin by collagen I matrix protein plays an important role in mediating human fetal PaSC adhesion, migration, proliferation, and growth factor production. Culturing human fetal PaSCs on collagen I not only enhanced α3β1 integrin expression, but also altered the integrin/FAK and downstream ERK and AKT signaling pathways, along with elevating cyclin D1 protein levels. Blocking β1 integrin on PaSCs resulted in significant down regulation of the FAK/ERK/cyclin D1 signaling pathways with no effect on the AKT pathway. This study provides the first *ex vivo* delineation of the mechanisms by which interaction of β1 integrin with collagen I protein regulates human fetal PaSC function and growth.

Using a modified PaSC isolation and purification protocol [14, 22], we showed that the stellate-like cells derived from the isolated human fetal pancreas expressed specific stellate markers including desmin, vimentin, GFAP, and αSMA, paired with the absence of CK19 and stromal cell markers, suggesting that ductal cells and mesenchymal stromal cells were not present as contaminants in isolated human PaSC populations [23-25]. More importantly, our purified human PaSCs retained functionality to produce various ECM and growth factors for at least 10 population doublings. Given this efficient method in isolating human fetal PaSCs, this study provided a sufficient cell number to elucidate PaSC function and PaSC-induced pancreatic diseases [26, 27], and also offers a novel opportunity to study PaSCs in pancreatic morphogenesis [10] and endocrine differentiation [28, 29] during pancreatic development.

To date there is insufficient available information regarding the expression of integrins on PaSCs in the developing human pancreas, although α5β1 integrin expression in rat adult PaSCs has been previously reported [11]. The present study examined the integrins associated with isolated human fetal PaSCs, and showed predominately high levels of α3β1 and α5β1. Examination of the relationship between ECM proteins and integrins required for human fetal PaSC adhesion and migration, demonstrated that collagen I plays a significant role through α3β1 integrin signaling. Culturing human fetal PaSCs on a collagen I matrix not only resulted in increased PaSC proliferation, but also significantly increased TGFβ1 and CTGF protein levels. Both TGFβ1 and CTGF are released by PaSCs and act upon PaSCs via autocrine signaling to control migration, proliferation, synthesis and secretion of ECM proteins [30-32]. Thus, increasing TGFβ1 and CTGF protein levels in cultured human PaSCs could promote a positive feedback loop to enhance cell adhesion, migration, and secretion in response to collagen I, specifically through α3β1 integrin. In contrast to human fetal PaSCs, α5β1 integrin is required for rat adult PaSC adhesion and migration on fibronectin under the CTGF stimulation [11], and blocking α5β1 integrin led to inhibited rat adult PaSC activation and function [33]. In hepatic stellate cells (HSCs), the interaction of β3 integrin with collagen I is required for HSC migration and survival, while inhibition of β3 integrin is sufficient for inducing HSC apoptosis and halting cell migration on collagen I matrix [34]. The differences in the integrin subunits and ECM interactions seen in the current PaSC study compared to others could be due to the cell types (HSCs *vs*. PaSCs), ages (fetal *vs*. adult), and species (human *vs*. rat). Our data demonstrated that the interaction between α3β1 integrin and the ECM protein collagen I is essential for the proliferation and function of human fetal PaSCs *in vitro*.

Importantly, the current findings were in agreement with previous reports showing that β1 integrin is predominately expressed in PaSCs [11]. Our previous study also showed that mice with β1 integrin deficiency under control of the collagen I promoter directly affected PaSC function and survival [10]. Using a neutralizing antibody to block β1 integrin activity on human fetal PaSCs, we found significantly inhibited cell adhesion, migration, proliferation, and growth factor production, along with reduced phosphorylation of the FAK/ERK and downstream cyclin D1 signaling that was independent of PI3K/AKT activation. In several model systems, β1 integrin has been shown to activate the FAK/ERK signaling cascade to enhance cell survival and function [35, 36]. Neutralizing antibodies against β1 and α3 integrin subunits could inhibit collagen I-induced activation of the FAK/ERK pathway in ovarian cancer cell lines [37]; however, enhanced activation of β1 integrin promotes phosphorylation of FAK, as seen in studies examining human lung fibroblasts viability [38-40]. Mantoni et al. [41] determined that PaSCs protect pancreatic cancer cells that had escaped from radiation through β1 integrin signaling via the FAK, but not the PI3K/AKT pathway. In addition, Lu et al. [42] found in their PaSC-induced cancer cell migration study that collagen I is the major factor to enhance α2β1 integrin expression, which lead to activation of the FAK signaling pathway. Our previous *in vitro* studies also demonstrated that collagen I promoted human fetal islet cell differentiation and proliferation through β1 integrin via downstream FAK/ERK phosphorylation [13], while only α3 integrin mediated signaling through the PI3K/AKT pathway [43]. Furthermore, β1 integrin signaling via the FAK/ERK pathway was verified in a mouse model with a collagen I-producing cell specific β1 integrin deficiency; these mice exhibited a severe loss of β-cell mass, suggesting that β1 integrin/FAK/ERK signaling is required for PaSC functions involved in β-cell differentiation and survival [10, 44]. Taken together, our present findings demonstrate that the β1 integrin/FAK/ERK signaling pathway is critical for human fetal PaSC function and survival.

In summary, the present study provides insight into the expression of integrin receptors in human fetal PaSCs and sheds light on how the β1 integrin, in association with its binding partner α3 subunit, interacts with the ECM protein collagen I and plays multiple roles in PaSC biology. β1 integrin interacts with the ECM to activate the FAK and ERK signaling pathway, which are important in maintaining proper PaSC function, along with increased cyclin D1 that leads to PaSC proliferation. These findings indicate that β1 integrin is required for the function and proliferation of human fetal PaSCs, and future applications of this research could contribute to the biomedical engineering of the ECM microenvironment needed for the efficient regulation of pancreatic development.

## MATERIALS AND METHODS

### Human fetal PaSCs isolation and culture

Human fetal pancreata (17-21 weeks fetal age) were collected according to protocols approved by the Health Sciences Research Ethics Board at the University of Western Ontario, in accordance with the Canadian Council on Health Sciences Research Involving Human Subjects guidelines. Pancreatic tissues were carefully dissected from surrounding tissues and immediately digested using dissociation buffer containing collagenase V (1 mg/ml; Sigma, St. Louis, MO, USA), followed by filtration through a 250 μm nylon mesh to yield fine cell-clusters [22]. PaSCs were isolated from dissociated human fetal pancreatic cell clusters using a modified outgrowth method as described previously [14]. In brief, cell clusters were seeded in uncoated culture T25 flasks (Fisher Scientific, Ottawa, ON, Canada) and cultured in Dulbecco's modified Eagle's medium (DMEM)/Ham's F12 (1:1 v/v) containing 10 % fetal bovine serum (FBS, Invitrogen, Burlington, ON, Canada). Eighteen hours after seeding, culture medium was changed with moving non-adhered cell clusters. The PaSCs grew out from the cell clusters over a 72 hours period. PaSC purity was assessed after the 2^nd^ passage by immunofluorescence staining and western blot analysis for stellate cell selective markers.

### Adhesion and migration assay

For the adhesion assay, 1×10^3^ PaSCs/well were plated on 96-well tissue culture plate pre-coated with 5μg/ml of either collagen I, collagen IV (BD Biosciences, Mississauga, ON, Canada), fibronectin, laminin (Chemicon, Temecula, CA, USA), or control (1% BSA, Sigma) in serum-free DEMEM/Ham's F12 (1:1 v/v) plus 1% BSA media for 20 minutes. Non-adhered cells were removed by washing the wells twice with sterile phosphate buffered saline (PBS). Six random fields per well were imaged at 40X magnification using an inverted light microscope (Leica DM IL; Leica Microsystems Inc., Concord, ON, Canada) and analyzed with Image ProPlus software (Media Cybernetics Inc., Rockville, MD, U.S.A). Adhered cells were counted manually, and data were expressed as fold change versus control.

To measure the migration rate of PaSCs, a wound-healing assay was adopted [45]. 1×10^4^ PaSCs/well were plated on 96-well tissue culture plate pre-coated with 5μg/ml of either collagen I, collagen IV, fibronectin, laminin, or control in serum-free media for 24 hours. After reaching confluence, the monolayers were wounded by scraping using a 10μl pipette tip and incubated with serum-free media for an additional 24 hours [45]. In each well, images of three different segments of the ‘wound’ area were captured 0 hour and 24 hours after damage. Each experiment was conducted in triplicate with at least four repeat experiments per group.

### Blocking of β1 integrin using immunoneutralizing antibody

PaSCs were pre-incubated with either anti-human β1 integrin antibody (CD29, 5μg/ml), human IgG2a isotype-matched negative control (5μg/ml, BD Biosciences, San Diego, CA, USA), or fresh media (DMEM/Ham's F12 plus 1% BSA) for 1 hour prior to seeding on collagen I (5μg/ml) pre-coated tissue culture plates [9, 12, 13, 43]. Cells were then cultured with DEMEM/Ham's F12 plus 1% BSA media for 24 hours. At the end of the culture period, cells were subjected to adhesion and migration assays, or harvested for protein preparation or immunocytochemical studies. All culture experiments were conducted in triplicate with at least 3 repeat experiments per group.

### Immunofluorescence staining

For in-situ cell staining, PaSCs were cultured on coverslips (Fisher Scientific) pre-coated with poly-L-lysine (Sigma) for 1-3 days. Immunofluorescence staining was conducted using the antibodies as listed in Table 1.

**Table 1:** List of antibodies used for immunofluorescence and/or western blotting analysis

Primary Antibody	Dilution	Company, Location
Mouse anti-α-Smooth Muscle Actin	1:50^a^ or 1:1000	Dako, Mississauga, ON, Canada
Mouse anti-Vimentin	1:200^a^ or 1:3000	Millipore, Temecula, CA, USA
Mouse anti-GFAP	1:50^a^ or 1:500	BD Pharmingen, Mississauga, ON, Canada
Rabbit anti-Desmin	1:100^a^ or 1:1000	Abcam, Cambridge, MA, USA
Mouse anti-collagen I	1:50^a^	Santa Cruz, Montreal, QC, Canada
Mouse anti-collagen IV	1:100^a^	Chemicon, Temecula, CA, USA
Rabbit anti-Laminin	1:100^a^	Developmental Studies Hybridoma Bank, Iowa City, IA, USA
Mouse anti-Fibronectin	1:100^a^	Chemicon, Temecula, CA, USA
Mouse anti-CK19	1:50^a^	Dako, Mississauga, ON, Canada
Mouse anti-Stromal cell surface marker	1:50^a^	Developmental Studies Hybridoma Bank, Iowa City, IA, USA
Mouse anti-β1	1:100^a^ or 1:1000	Chemicon, Temecula, CA, USA
Rabbit anti-β3	1:50^a^	Abcam, Cambridge, MA, USA
Rabbit anti-α1	1:50^a^	Santa Cruz, Montreal, QC, Canada
Rabbit anti-α2	1:100^a^ or 1:2000	Santa Cruz, Montreal, QC, Canada
Rabbit anti-α3	1:200^a^or 1:2000	Chemicon, Temecula, CA, USA
Rabbit anti-α5	1:500^a^or 1:5000	Chemicon, Temecula, CA, USA
Rabbit anti-αV	1:200^a^or 1:2000	Santa Cruz, Montreal, QC, Canada
Mouse anti-Ki67	1:100^a^	BD Pharmingen, Mississauga, ON, Canada
Mouse anti-phosphorylated Akt (Ser 473)	1:2000	Cell Signaling, Danvers, MA, USA
Rabbit anti-Akt	1:2000	Cell Signaling, Danvers, MA, USA
Rabbit anti-phosphorylated Thr202/Tyr204 ERK12	1:2000	Cell Signaling, Danvers, MA, USA
Rabbit anti-ERK1/2	1:1000	Cell Signaling, Danvers, MA, USA
Rabbit anti-phosphorylated FAK (Try397)	1:2000	Abcam, Cambridge, MA, USA
Rabbit anti-FAK	1:3000	Cell Signaling, Danvers, MA, USA
Mouse anti-CyclinD1	1:2000	Cell Signaling, Danvers, MA, USA
Rabbit anti-TGF-β1	1:1000	Sigma, St Louis, MO, USA
Goat anti-CTGF	1:1000	Santa Cruz, Montreal, QC, Canada
Mouse anti-Calnexin	1:2000	BD Biosciences, Mississauga, ON, Canada
Rabbit anti-GAPDH	1:2000	Santa Cruz, Montreal, QC, Canada

a dilution factor used for immunofluorescence.

For quantitative analysis of Ki67 labeling in PaSCs, experimental cell groups at the end of the culture were harvested and fixed in 4% PFA, embedded in 2% agarose gel and processed into tissue blocks [43]. Cell sections (4μm) were stained for Ki67 and nuclei were counterstained with DAPI. The percentage of Ki67^+^ cells was calculated by counting at least 500 cells per section per experimental group, with a minimum of 3 repeat experiments per group.

### Protein extraction and western blotting

Protein from PaSCs was extracted in a NP-40 lysis buffer. Equal amount of protein from each experimental group was fractionated by 7.5% or 10% sodium dodecyl sulfate-polyacrylamide gel electrophoresis (SDS-PAGE) and transferred onto a nitrocellulose membrane (Bio-Rad Laboratories, Mississauga, ON, Canada). Membranes were incubated with primary antibodies of appropriate dilution as listed in Table 1, followed by the application of appropriate horse radish peroxidase (HRP)-conjugated secondary antibodies. Proteins were detected using ECL™-Plus Western blot detection reagents (Perkin Elmer, Wellesley, MA, USA) and imaged by the Versadoc Imaging System (Bio-Rad Laboratories). Bands were densitometrically quantified by Image Lab 3.0 software (Bio-Rad Laboratories) and normalized to appropriate loading controls [9, 13, 43].

### Statistical analysis

Data are expressed as mean ± SEM. Statistical significance was determined using the paired Student's *t-*test when comparing two groups, or one-way ANOVA followed by Tukey's and/or Fisher's least significant difference post-hoc test when comparing more than two groups using GraphPad Prism software program (Version 5.0c, GraphPad Software Inc., La Jolla, CA, USA) or SPSS software (version 19.0, SPSS Inc, Chicago, IL, USA). Differences were considered to be statistically significant when *p* < 0.05.

## References

[1] Phillips PA, Yang L, Shulkes A, Vonlaufen A, Poljak A, Bustamante S, Warren A, Xu Z, Guilhaus M, Pirola R, Apte MV, Wilson JS (2010). Pancreatic stellate cells produce acetylcholine and may play a role in pancreatic exocrine secretion. Proc Natl Acad Sci USA.

[2] Kordes C, Sawitza I, Götze S, Häussinger D (2012). Stellate Cells from Rat Pancreas Are Stem Cells and Can Contribute to Liver Regeneration. PLoS One.

[3] Murray IR, West CC, Hardy WR, James AW, Park TS, Nguyen A, Tawonsawatruk T, Lazzari L, Soo C, Péault B (2014). Natural history of mesenchymal stem cells, from vessel walls to culture vessels. Cell Mol Life Sci.

[4] Saotome T, Inoue H, Fujimiya M, Fujiyama Y, Bamba T (1997). Morphological and immunocytochemical identification of periacinar fibroblast-like cells derived from human pancreatic acini. Pancreas.

[5] Haber PS, Keogh GW, Apte MV, Moran CS, Stewart NL, Crawford DH, Pirola RC, McCaughan GW, Ramm GA, Wilson JS (1999). Activation of pancreatic stellate cells in human and experimental pancreatic fibrosis. Am J Pathol.

[6] Hynes RO (2002). Integrins: bidirectional, allosteric signaling machines. Cell.

[7] Brakebusch C, Fässler R (2005). beta 1 integrin function *in vivo*: adhesion, migration and more. Cancer Metastasis Rev.

[8] Juliano RL, Reddig P, Alahari S, Edin M, Howe A, Aplin A (2004). Integrin regulation of cell signalling and motility. Biochem Soc Trans.

[9] Krishnamurthy M, Li J, Al-Masri M, Wang R (2008). Expression and function of alphabeta1 integrins in pancretic beta (INS-1) cells. J Cell Commun Signal.

[10] Riopel MM, Li J, Liu S, Leask A, Wang R (2013). β1 integrin-extracellular matrix interactions are essential for maintaining exocrine pancreas architecture and function. Lab Invest.

[11] Gao R, Brigstock DR (2005). Connective tissue growth factor (CCN2) in rat pancreatic stellate cell function: integrin alpha5beta1 as a novel CCN2 receptor. Gastroenterology.

[12] Wang R, Li J, Lyte K, Yashpal NK, Fellows F, Goodyer CG (2005). Role for beta1 integrin and its associated alpha3, alpha5, and alpha6 subunits in development of the human fetal pancreas. Diabetes.

[13] Saleem S, Li J, Yee SP, Fellows GF, Goodyer CG, Wang R (2009). beta1 integrin/FAK/ERK signalling pathway is essential for human fetal islet cell differentiation and survival. J Pathol.

[14] Bachem MG, Schneider E, Gross H, Weidenbach H, Schmid RM, Menke A, Siech M, Beger H, Grünert A, Adler G (1998). Identification, culture, and characterization of pancreatic stellate cells in rats and humans. Gastroenterology.

[15] Coppolino M, Migliorini M, Argraves WS, Dedhar S (1995). Identification of a novel form of the alpha 3 integrin subunit: covalent association with transferrin receptor. Biochem J.

[16] Delwel GO, de Melker AA, Hogervorst F, Jaspars LH, Fles DL, Kuikman I, Lindblom A, Paulsson M, Timpl R, Sonnenberg A (1994). Distinct and overlapping ligand specificities of the alpha 3A beta 1 and alpha 6A beta 1 integrins: recognition of laminin isoforms. Mol Biol Cell.

[17] Fogerty FJ, Akiyama SK, Yamada KM, Mosher DF (1990). Inhibition of binding of fibronectin to matrix assembly sites by anti-integrin (alpha 5 beta 1) antibodies. J Cell Biol.

[18] Pankov R, Cukierman E, Katz BZ, Matsumoto K, Lin DC, Lin S, Hahn C, Yamada KM (2000). Integrin dynamics and matrix assembly: tensin-dependent translocation of alpha(5)beta(1) integrins promotes early fibronectin fibrillogenesis. J Cell Biol.

[19] Kusano Y, Oguri K, Nagayasu Y, Munesue S, Ishihara M, Saiki I, Yonekura H, Yamamoto H, Okayama M (2000). Participation of syndecan 2 in the induction of stress fiber formation in cooperation with integrin alpha5beta1: structural characteristics of heparan sulfate chains with avidity to COOH-terminal heparin-binding domain of fibronectin. Exp Cell Res.

[20] Apte MV, Haber PS, Darby SJ, Rodgers SC, McCaughan GW, Korsten MA, Pirola RC, Wilson JS (1999). Pancreatic stellate cells are activated by proinflammatory cytokines: implications for pancreatic fibrogenesis. Gut.

[21] Karger A, Fitzner B, Brock P, Sparmann G, Emmrich J, Liebe S, Jaster R (2008). Molecular insights into connective tissue growth factor action in rat pancreatic stellate cells. Cell Signal.

[22] Vonlaufen A, Phillips PA, Yang L, Xu Z, Fiala-Beer E, Zhang X, Pirola RC, Wilson JS, Apte MV (2010). Isolation of quiescent human pancreatic stellate cells: a promising *in vitro* tool for studies of human pancreatic stellate cell biology. Pancreatology.

[23] Apte MV, Pirola RC, Wilson JS (2012). Pancreatic stellate cells: a starring role in normal and diseased pancreas. Front Physiol.

[24] Omary MB, Lugea A, Lowe AW, Pandol SJ (2007). The pancreatic stellate cell: a star on the rise in pancreatic diseases. J Clin Invest.

[25] Zha M, Li F, Xu W, Chen B, Sun Z (2014). Isolation and characterization of islet stellate cells in rat. Islets.

[26] Kikuta K, Masamune A, Hamada S, Takikawa T, Nakano E, Shimosegawa T (2013). Pancreatic stellate cells reduce insulin expression and induce apoptosis in pancreatic β-cells. Biochem Biophys Res Commun.

[27] Masamune A, Shimosegawa T (2013). Pancreatic stellate cells—multi-functional cells in the pancreas. Pancreatology.

[28] El-Gohary Y, Tulachan S, Guo P, Welsh C, Wiersch J, Prasadan K, Paredes J, Shiota C, Xiao X, Wada Y, Diaz M, Gittes G (2013). Smad signaling pathways regulate pancreatic endocrine development. Dev Biol.

[29] Guney MA, Petersen CP, Boustani A, Duncan MR, Gunasekaran U, Menon R, Warfield C, Grotendorst GR, Means AL, Economides AN, Gannon M (2011). Connective tissue growth factor acts within both endothelial cells and beta cells to promote proliferation of developing beta cells. Proc Natl Acad Sci USA.

[30] Aoki H, Ohnishi H, Hama K, Ishijima T, Satoh Y, Hanatsuka K, Ohashi A, Wada S, Miyata T, Kita H, Yamamoto H, Osawa H, Sato K (2006). Autocrine loop between TGF-beta1 and IL-1beta through Smad3- and ERK-dependent pathways in rat pancreatic stellate cells. Am J Physiol Cell Physiol.

[31] Shek FW, Benyon RC, Walker FM, McCrudden PR, Pender SLF, Williams EJ, Johnson CD, Bateman AC, Fine DR, Iredale JP (2002). Expression of transforming growth factor-beta 1 by pancreatic stellate cells and its implications for matrix secretion and turnover in chronic pancreatitis. Am J Pathol.

[32] Fitzner B, Brock P, Nechutova H, Glass A, Karopka T, Koczan D, Thiesen HJ, Sparmann G, Emmrich J, Liebe S, Jaster R (2007). Inhibitory effects of interferon-gamma on activation of rat pancreatic stellate cells are mediated by STAT1 and involve down-regulation of CTGF expression. Cell Signal.

[33] Gao R, Brigstock DR (2006). A novel integrin alpha5beta1 binding domain in module 4 of connective tissue growth factor (CCN2/CTGF) promotes adhesion and migration of activated pancreatic stellate cells. Gut.

[34] Zhou X, Murphy FR, Gehdu N, Zhang J, Iredale JP, Benyon RC (2004). Engagement of alphavbeta3 integrin regulates proliferation and apoptosis of hepatic stellate cells. J Biol Chem.

[35] Choma DP, Milano V, Pumiglia KM, DiPersio CM (2007). Integrin alpha3beta1-dependent activation of FAK/Src regulates Rac1-mediated keratinocyte polarization on laminin-5. J Invest Dermatol.

[36] Chen CA, Tsai JC, Su PW, Lai YH, Chen HC (2006). Signaling and regulatory mechanisms of integrinalpha3beta1 on the apoptosis of cultured rat podocytes. J Lab Clin Med.

[37] Ahmed N, Riley C, Rice G, Quinn M (2005). Role of integrin receptors for fibronectin, collagen and laminin in the regulation of ovarian carcinoma functions in response to a matrix microenvironment. Clin Exp Metastasis.

[38] Tian B, Lessan K, Kahm J, Kleidon J, Henke C (2002). beta 1 integrin regulates fibroblast viability during collagen matrix contraction through a phosphatidylinositol 3-kinase/Akt/protein kinase B signaling pathway. J Biol Chem.

[39] Xia H, Nho RS, Kahm J, Kleidon J, Henke CA (2004). Focal adhesion kinase is upstream of phosphatidylinositol 3-kinase/Akt in regulating fibroblast survival in response to contraction of type I collagen matrices via a beta 1 integrin viability signaling pathway. J Biol Chem.

[40] Nho RS, Xia H, Kahm J, Kleidon J, Diebold D, Henke CA (2005). Role of integrin-linked kinase in regulating phosphorylation of Akt and fibroblast survival in type I collagen matrices through a beta1 integrin viability signaling pathway. J Biol Chem.

[41] Mantoni TS, Lunardi S, Al-Assar O, Masamune A, Brunner TB (2011). Pancreatic stellate cells radioprotect pancreatic cancer cells through β1-integrin signaling. Cancer Res.

[42] Lu J, Zhou S, Siech M, Habisch H, Seufferlein T, Bachem MG (2014). Pancreatic stellate cells promote hapto-migration of cancer cells through collagen I-mediated signalling pathway. Br J Cancer.

[43] Krishnamurthy M, Li J, Fellows GF, Rosenberg L, Goodyer CG, Wang R (2011). Integrin {alpha}3, but not {beta}1, regulates islet cell survival and function via PI3K/Akt signaling pathways. Endocrinology.

[44] Riopel M, Krishnamurthy M, Li J, Liu S, Leask A, Wang R (2011). Conditional β1-integrin-deficient mice display impaired pancreatic β cell function. J Pathol.

[45] Li L, Bimmler D, Graf R, Zhou S, Sun Z, Chen J, Siech M, Bachem MG (2011). PSP/reg inhibits cultured pancreatic stellate cell and regulates MMP/ TIMP ratio. Eur J Clin Invest.

